# Genome Editing Fidelity in the Context of DNA Sequence and Chromatin Structure

**DOI:** 10.3389/fcell.2020.00319

**Published:** 2020-05-08

**Authors:** Lyuba Chechik, Ophelie Martin, Evi Soutoglou

**Affiliations:** ^1^Institut de Génétique et de Biologie Moléculaire et Cellulaire, Illkirch, France; ^2^Institut National de la Santé et de la Recherche Médicale, U964, Illkirch, France; ^3^Centre National de Recherche Scientifique, UMR 7104, Illkirch, France; ^4^Université de Strasbourg, Strasbourg, France; ^5^Genome Damage and Stability Centre, School of Life Sciences, University of Sussex, Brighton, United Kingdom

**Keywords:** chromatin, dna editing crispr, knock in, DNA repair, nucleus

## Abstract

Genome editing by Clustered Regularly Inter Spaced Palindromic Repeat (CRISPR) associated (Cas) systems has revolutionized medical research and holds enormous promise for correcting genetic diseases. Understanding how these Cas nucleases work and induce mutations, as well as identifying factors that affect their efficiency and fidelity is key to developing this technology for therapeutic uses. Here, we discuss recent studies that reveal how DNA sequence and chromatin structure influences the different steps of genome editing. These studies also demonstrate that a deep understanding of the balance between error prone and error free DNA repair pathways is crucial for making genome editing a safe clinical tool, which does not induce further mutations to the genome.

## Introduction

Genome editing is very valuable for both medical and research purposes. Future medical applications include the correction of disease-related mutations, disruption of disease-promoting genes or even introducing novel genes (e.g., for sensitising immune system to tumour cells). Research applications range from creating knock-out/knock in cell line or organisms, and/or introducing mutations, to study the role of a particular protein, pathway or processes to creating humanized disease models. Given the tempting scope of practical use, it is of no surprise that there has been considerable effort in developing genome editing methods. The traditional way for introducing changes to the genome was by the use of spontaneous recombination, either to introduce DNA mutations or to insert sequences that would allow further use of recombinases (such as Cre) to excise genes [reviewed in Sauer (2002)]. Subsequent discoveries of zinc finger nucleases (ZFNs) and transcription activator-like effector nucleases (TALENs) allowed a considerable advance in the field by allowing the introduction of DNA breaks at desired, rather than random, genomic locations [reviewed in Gaj et al. (2013)]. Nevertheless, the biggest advance in genome editing has been the more recent discovery of clustered regularly interspaced palindromic repeat (CRISPR) associated (Cas) systems (Ishino et al., 1987; Jansen et al., 2002; Jinek et al., 2012; Cong et al., 2013; Mali et al., 2013).

Shortly after its discovery, the CRISPR-Cas9 system, a bacterial defense mechanism, was repurposed as a powerful tool for genome editing in plant, animal and human cells due to its specificity and its easier implementation. Current and future potential uses cover a wide range of application in research and clinical areas, by allowing substitution, insertion or deletion to the DNA sequence in a targeted genomic location [reviewed in Hsu et al. (2014) and Wang and Qi (2016)]. The CRISPR-Cas9 system operates through the recruitment of the RNA-guided Cas9 nuclease at a specific genomic position. The targeting relies on the complementarity between the guide RNA and the targeted sequences and the presence of an adjacent DNA protospacer motif (PAM). The Cas9 nuclease generates a DNA double strand break (DSB) at the targeted sequence adjacent to the PAM sequence (Jiang and Doudna, 2017), which then leads to recruitment of DNA repair machinery to fix the break.

Typically, DNA DSBs are repaired by (i) the error free homologous recombination (HR) pathway, which occurs in S/G2 phases of the cell cycle as it uses the homologous sequences of the sister chromatids as a repair template, and (ii) the error prone non-homologous end joining (NHEJ) pathway, which occurs throughout the cell cycle and religates DNA ends without the presence of an undamaged template (Ciccia and Elledge, 2010). In addition, other alternative end joining pathways, which rely on the presence of microhomologies (MH mediated end joining, MMEJ), have been described, these DSB repair pathways are error prone and are often associated with long deletions (Decottignies, 2013; Chang et al., 2017).

DNA end resection is a major determinant influencing DNA repair pathway choice. Unresected DNA ends, processed by the NHEJ pathway, are bound to the Ku complex (Ku70-Ku80 heterodimer) which recruits NHEJ factors including DNA-PKcs (DNA dependent protein kinase catalytic subunit), XRCC4 (X ray repair cross complementing 4) and LIG IV (DNA ligase IV) to catalyze DNA ends ligation. In contrast, the MMEJ pathway requires minimal DNA ends resection (through the CtIP-MRN complex) that reveals homologies on opposite strands that will be further involved in annealing. DNA portion between homologies is removed, leading to deletion scars. Other MMEJ factors are further recruited to resolve the break, including DNA polymerase θ (POL Q), and the DNA ligases I and III (Decottignies, 2013; Chang et al., 2017).

DNA repair pathway choice is regulated at different levels: cell cycle stage, availability and post translational modifications of DNA repair factors, chromatin status and the position within the nucleus of the break [reviewed in Kalousi and Soutoglou (2016)]. The choice of pathway can have critical consequences for the cell, since the use of error prone pathways can lead to unwanted deleterious mutations. Despite the many efforts put into characterizing repair pathways, Cas9-induced DSB repair outcomes have not been yet extensively investigated. It is crucial, for both research and clinical purposes, to precisely understand how mutation profiles observed following Cas9-induced DSB are generated, to be able to predict repair outcomes. In this review, we will focus on recent work highlighting the outcome of CRISPR-Cas9-induced DSBs in mammalian cells. Interestingly, the CRISPR-Cas9 mutational pattern appears to be non-random, highly reproducible and mainly dependent on the targeted DNA sequence.

## Cas 9-Mutational Profiles Are Largely Dependent on the Target DNA Sequence

Several studies have revealed the prominent role of the target DNA sequence in Cas9-dependent DNA repair outcomes. In these studies, repair outcomes were profiled by classifying the mutations generated at Cas9 target sites by the type of insertion or deletion (indel) that occurred (e.g., size, position, microhomology), and monitoring the frequency of each class of indel. van Overbeek et al. (2016) were the first to conduct a systematic study of DNA repair profiles following Cas9 cleavage in human cell lines. They followed the repair outcomes after guide RNAs delivery targeting 69 different genomic sites and demonstrated that indel patterns differed from one targeted site to another and were very reproducible among replicates and between cell types. Nevertheless, the mutation frequencies of a given indel class varied with cell type. Taken together, this suggests that the characteristic DNA repair profile associated with a genomic location is influenced by the DNA sequence around the targeted area (van Overbeek et al., 2016). To further confirm this conclusion, guide RNAs matching multiple locations in human genome (“multiple target single spacers,” MTSS) were designed and the associated indel profiles were assessed. In line with their previous observations, similarities between repair profiles for each site targeted by the same guide RNA are observed across replicates and cell type (van Overbeek et al., 2016).

Allen et al. (2018) confirmed such observations by specifically interrogating the influence of the DSB-flanking DNA sequence on repair outcomes. The authors designed and delivered synthetic constructs containing both a guide RNA and its target sequence flanked by variable DNA sequences, in human K562 cells. Indel profile analysis revealed that indels were highly reproducible and sequence-specific. Moreover, shorter deletions were more prominent compared to longer deletions, with nucleotide insertions (+1) and deletions (-1) being the most common. 58% of all Cas9-generated deletions, however, were at least 3 bp long and about a half of them occurred between at least two nucleotide repeats, referred to as microhomology (MH). The deletion frequency resulting from MH presence was inversely correlated with the distance between MH sequences. Introducing point mutation(s) in MH regions led to a remarkable drop in the associated repair outcome frequency (Allen et al., 2018). Intriguingly, although the indel patterns were similar across most cell types, stem cells had more large deletions and MH mediated products, whereas single nucleotide insertions (+1 insertions) were more frequent in differentiated cells. It was proposed that such observations correlate with different activities for the DNA repair pathways in different cell types.

Furthermore, indel profiling revealed that for almost half (49%) of the guide RNAs with a T (thymidine) before the cut site, a + 1 insertion involving another T dominates the repair outcome. A bias was also observed regarding small deletions: 77% of -1 deletions are associated with the removal of a repeated nucleotide at the break site. For half of the dinucleotide deletions, the removal of a two- base repeat was also quite common (Allen et al., 2018). These results are in agreement with Lemos et al. (2018), who demonstrated that single base insertions were shown to preferably repeat a PAM-distal nucleotide at the break site in yeast.

A recent large-scale study shed further light on the influence of genetic and epigenetic factors in CRISPR-Cas9 repair outcomes (Chakrabarti et al., 2019). Analysis of indel patterns at approximately 1,500 targeted locations in human cells (HepG2), revealed again that DNA editing precision differs across sites in a non-random and reproducible manner. The majority of examined targeted sites showed a preference for small indels (44% for 1 bp insertion and 26% for 1 bp deletion). However, a preference for large deletions (up to 41 nucleotides) was also observed for some sites. As a consequence of single nucleotide modifications, a considerable bias toward frameshifting mutations was observed (average of 80.1% compared to 66% of a random outcome).

Editing precision (recurrence of a specific indels) varied considerably between different targets with some targets associated with a large number (up to 79) of distinct, infrequent, deletions. In contrast, other targets showed one dominant mutation (representing up to 94% of all repair events). Overall, one fifth of all analyzed targets had at least a 50% chance of leading to a specific indel. Based on the distribution of commonest indel frequencies, the targeted sites were categorized into three groups: imprecise (commonest indel frequency below 25%), middle (commonest indel frequency below 50%), and precise (commonest indel frequency above 50%) sites. The vast majority of recurrent indels in precise targets (68.4%) are associated with a strong preference for insertions with a bias toward single nucleotide indels. In agreement with Allen et al. insertion, of a single nucleotide homologous to a PAM distal nucleotide (at position -4) at the break site was very common, especially when this nucleotide is T. These observations are consistent with Taheri-Ghahfarokhi et al. (2018), who also highlighted the importance of the 4th nucleotide before the PAM in the single nucleotide indel frequencies.

Strikingly, not only the indel pattern but also the editing precision could be predicted from the target site DNA sequence. Using a neural network Chakrabarti et al. found a significant correlation between the computational (estimated) and the observed indel frequencies. Despite a moderate predictive power of the model, it allowed the identification of key sequencing features. This computational quest also led to the conclusion that the nucleotide at position -4 from the PAM strongly influences the repair outcome in accordance with all previous experimental observations.

All in all, both by experimental studies and computer simulations, the Cas9-associated indel pattern and a presence of a dominant pattern appear to be mostly dependent on the DNA sequence around a break site, with the presence of MH in the target DNA sequence one of the main cues for predictability.

## Cas 9-Mutational Profiles Rely On MMEJ

The types of indel observed upon CRISPR-Cas9 cleavage suggest that Cas9-induced breaks are mainly repaired by NHEJ and MMEJ. It is generally assumed that small indels (<3 bp) occur via NHEJ and longer deletions occur via MMEJ. When analyzing the indel distribution following CRISPR-Cas9 activity over for a 48 h period, van Overbeek et al. showed that larger deletions are more prevalent at later points. They also observed that upon inhibition of NHEJ, +1 insertions and small indel (<3 bp) frequencies were decreased and, in contrast, large deletions (>3 bp) frequencies were increased (van Overbeek et al., 2016). The fact that alteration of NHEJ leads to increased MMEJ usage points to a tight balance between NHEJ and MMEJ pathways in repairing these breaks. Similar studies were performed later by Brinkman et al. for a single locus in human K562 cells. Targeting the *LBR* locus, the indel pattern analysis revealed a +1 insertion in balance with a -7 bp deletion. Addition of the NHEJ inhibitor NU7441 led to an increase of -7 deletions concomitant to a decrease in +1 insertions. Addressing the kinetics of the two processes revealed that MMEJ is delayed and initiated after NHEJ, and the delay is not observed when NHEJ is inhibited arguing for MMEJ predominantly being used as a back-up to repair breaks that, for unknown reason, failed to engage NHEJ (Brinkman et al., 2018).

Aiming to characterize in detail the contribution of the MMEJ pathway in the repair outcomes following Cas9 activity, Taheri et al. developed a computational platform called RIMA (Rational Indel Meta Analysis). Two datasets from the literature were reanalyzed using RIMA to validate their approach. They confirmed MMEJ pathway involvement in DNA repair after Cas9 cleavage and MMEJ-associated indels enrichment upon NU7441 (Bae et al., 2014; van Overbeek et al., 2016; Taheri-Ghahfarokhi et al., 2018). They also confirmed that larger indels and other MMEJ events relied on the activity of the known MMEJ factor POLQ (Taheri-Ghahfarokhi et al., 2018).

Experiments to determine the contribution of MH to the CRISPR-cas9 dependent DNA repair outcome by Chakrabarti et al. revealed that microhomologies of different sizes were responsible for a majority of deletions (73.3%). Strikingly, deletions associated with short microhomologies (1–4 bp), typically not considered as a substrate for MMEJ, were also enriched indicating a role for homology regions of any length MH, not restricted only to long regions of MH as had previously been believed (Chakrabarti et al., 2019). In line with these observations, Bae et al. found that a large subset of all observed deletions upon Cas9 activity were associated with 2–8 bp MH sequences. Based on this observation, the authors developed a computer program to predict MH-dependent deletions at a given site in order to increase the frequency of gene disruption (Bae et al., 2014).

Despite how incomplete our understanding of the exact role of MH involvement in the repair process is, it has already been flagged for its potential practical applications. In their recent work, Kim et al. demonstrated the possibility of using this genomic feature for obtaining a desired genome editing effect. They suggested an elegant two-step scheme for introducing point mutations in human iPS cells, associated with scar-less selection marker excision. Initially the desired mutation is introduced into the locus of interest as engineered MH sequences flanking a selection marker used as a donor. Although positive selection based on the presence of the selective marker represents an easy way to obtain clonal population, some applications require the removal of the selective marker. Therefore after positive selection, the selection marker can be excised using CRISPR-Cas9 induced DSBs targeting the region adjacent to the MH sequences, promoting the use of MMEJ for the selection marker excision while preserving the point mutation (Kim et al., 2018).

Overall, based on both computational and experimental studies, MH arises as a major factor influencing the DNA repair outcome at CRISP-Cas9 lesions. However, whether it is indeed an underestimated role of the MMEJ pathway or a lack of a deep understanding of NHEJ pathway functioning remains to be seen.

## Cas 9-Mediated Large Deletions and Complex Repair Outcomes

Most of the studies addressing repair of Cas9-induced breaks were focused on deletions of a relatively small size, based on the belief that NHEJ and MMEJ are the main pathways involved. However, large-scale indel pattern analysis highlights the complexity of Cas9-dependent repair outcomes. Such complexity is well depicted in the Shin et al. study where they analyzed the consequence of CRISPR-Cas9-mediated genome editing in founder mice (Shin et al., 2017). They showed that the majority of detected deletions were asymmetric (1.5-fold or more difference between deletion up-and downstream of the cutting site). Prevalence of asymmetric indels was observed for almost all targeted sites. Symmetric deletions were infrequent and tended to be small (less than 10 bp). Moreover, the deletions mostly occurred at repetitive regions, which is consistent with the conclusions of the above-mentioned studies relating to the role of MH in DSB repair.

Induction of DSBs with single guide RNAs in murine zygotes also revealed a 9 bp median deletion size, but larger deletions (up to 600 bp) were also present (Kim et al., 2018).

Testing whether sequential or simultaneous guide RNAs delivery would have any effect on an indel pattern and on a balance between small and large deletions, revealed that sequential guide RNAs delivery is more reliable than simultaneous in precisely deleting juxtaposed sites. Moreover, while no difference was observed for smaller deletions (less than 400 bp) between the two delivery strategies, deletions larger than 400 bp (up to 24 kb) were only present after simultaneous delivery. These large deletions didn’t appear to rely on the presence of MH (Kim et al., 2018).

In light of the potential therapeutic use of Cas9, the findings of Kosicki et al., 2018 are especially striking. The authors explored large genetic alterations observed after CRISPR-Cas9 activity, focusing primarily on large deletions, which often are missing from repair outcome analysis due to a strong focus on a region proximal to the break (Kosicki et al., 2018). They performed knock-out experiments in mESC with single guide RNAs and observed that more than 20% of resulting alleles carried large (>250 bp and up to 6 kb) deletion. Even more surprisingly, in more than 15% of cases they observed additional DNA alterations (point mutations, large or small indels), distal to the cut site. Large inversions and duplications were also observed. Using mESCs obtained from a cross between two murine strains, Kosicki et al. also observed cases of loss of heterozygosity, presumably caused by using a homologous chromosome as a template. Despite differences in indel profile frequencies observed between stem cells and differentiated cells (Allen et al., 2018), larger deletions are not a unique feature associated with stem cells since they were observed in mouse hematopoietic progenitors cells and human RPE-1 cells (Kosicki et al., 2018).

Together, these data suggest, Cas9-mediated genome editing appears to be more complex and involves larger genome regions than was thought before. Thus, it is extremely important to understand the reasons for such an effect, and to take this into account while assessing using Cas9 for any medical purpose.

## Chromatin Structure Influences Cas 9 Binding

The chromatin structure around DNA breaks influences DNA repair pathway choice (Kalousi and Soutoglou, 2016). However, regarding the repair of Cas9-mediated breaks, the question arises; which step of Cas9 editing (binding, cutting and/or repair) is most influenced by chromatin state? To dissect this, some *in vitro* and *in vivo* studies have been performed. First, Isaac et al. developed a biochemical assay to determine how nucleosomes and chromatin remodellers influence Cas9 activity. Using nucleosome assembly associated with poor breathing (a term that defines the dynamic binding of histones to DNA), they observed that Cas9 binding activity and cutting is inhibited. In contrast, Cas9-induced cleavage is achieved near to the entry/exit of a nucleosome assembly associated with higher breathing. Furthermore, the authors demonstrated that different classes of chromatin remodellers enhanced Cas9 activity, with an increase of Cas9-mediated cleavage in the presence of remodellers from the ISWI family promoting nucleosome sliding (SNF2h) or histone octamer eviction (RSC) (Isaac et al., 2016).

At the same time, a study conducted by Horlbeck et al. led to the same observations *in vivo* and *in vitro*. The authors first overlaid data obtained from a CRISPR screen (Gilbert et al., 2015) with MNase-seq experiments publicly available at ENCODE (performed in K562 human cells) and observed that high nucleosome occupancy is associated with low CRISPR interference activity (for CRISPR interference, catalytically inactive Cas9 is fused to a transcriptional repressor and guided to the targeted site in order to interfere with gene transcription) (Horlbeck et al., 2016). Along similar lines, *in vitro* experiments argued for a block of Cas9 activity in the presence of DNA assembled into nucleosomes (Hinz et al., 2015; Horlbeck et al., 2016). Using an inducible system to control chromatin state (open or close) in human cells at a specific locus, Daer et al. observed reduced editing efficiency associated with heterochromatin (closed state) due to a reduction in Cas9 binding, for six over a total of nine guide RNAs used. This observation suggests that the effect of closed chromatin on Cas9 editing is guide RNA dependent or that in such inducible system the closed chromatin spreading is not covering equally all targeted sequences. Nevertheless, the mutation signature was not affected by the chromatin state. Interestingly, editing efficiency could be restored by artificial transcription activation (Daer et al., 2017).

Cas9 binding has also been studied in ChIP experiments in mouse ESC in which catalytically inactive Cas9 (dead Cas9) has been expressed. These studies also revealed that chromatin accessibility (assessed by DNAse I hypersensitivity experiments) is an important determinant of Cas9 binding *in vivo* and the vast majority of Cas9 off target sites are associated with active genes (Wu et al., 2014). Such findings were later confirmed by Kuscu et al. (2014) and O’Geen et al. (2015) that demonstrated a correlation between open chromatin and Cas9 off target binding in human and mouse cell lines, respectively.

Thus there is a general agreement that Cas9 activity is influenced by chromatin structure both *in vivo* and *in vitro*, with closed chromatin associated with less Cas9 binding and editing.

## The Role of Chromatin in Cas 9-Mediated Genome Editing

The degree of influence of chromatin state over Cas9-induced mutagenesis has been the subject of studies by several research teams over the last few years. Chen et al. interrogated how chromatin status influences TALEN and CRISPR-Cas9 genome editing activity. For this purpose, a cellular system carrying a reporter in which chromatin status can be switched from compacted (H3K9me3 marked) to relaxed was used. Lower editing efficiency was observed when targeted sites were associated with heterochromatin for both TALENs and Cas9 nucleases, but the impact of chromatin state on editing was higher for TALENs. Interestingly, the efficiency of DSB formation was quite comparable (Chen et al., 2016). Subsequently, Chen et al. assessed the influence of chromatin structure on Cas9 editing in whole organisms. Zebrafish embryos were co-injected with guide RNAs and Cas9 mRNA. Editing efficiency positively correlated with chromatin accessibility (determined by ATAC-seq), and mutation rates were higher in an open chromatin. However, there was no correlation between nucleosome-occupancy and editing efficiency (Chen et al., 2017). The latter can be explained by high nucleosome dynamics in early zebrafish embryos, which is in line with the observations of Isaac et al., 2016 that pointed out that Cas9 activity is influenced by nucleosome breathing (Isaac et al., 2016). A study conducted by Kallimasioti-Pazi et al. induced Cas9 breaks at three different imprinted genes in mESC and demonstrated a delayed accumulation of mutations in heterochromatin compared to euchromatin. The allele-specific editing bias toward the active allele was particularly apparent in the case of low Cas9 expression or short Cas9 expression periods. In cells in which imprinting at the targeted locus had been lost, due to prolonged culture, there was a restoration of Cas9 editing efficiency, which again implies an heterochromatic environment impairs editing (Kallimasioti-Pazi et al., 2018). It does not appear to be the DNA methylation status of heterochromatin that is responsible for affecting cas9-mediated break editing, since Hsu et al. demonstrated that Cas9 mediated cleavage is not affected by CpG DNA methylation as supported by indel detection (around 8%) at the silent highly methylated SERPINB5 targeted locus (Hsu et al., 2013).

Kallimasioti-Pazi et al. (2018) could detect by allele-specific ChIP, that Cas9 binding was lower in heterochromatin, which correlated with the slowed rate of mutagenesis, thus confirming conclusions of Isaac et al. (2016) and Daer et al. (2017). Interestingly, despite distinct epigenetic statuses, the same mutation pattern was observed on maternal or paternal alleles arguing for an influence of heterochromatin on the kinetics but not on the outcome of Cas9 editing (Kallimasioti-Pazi et al., 2018). In line with such observations, using live cell single-molecule tracking in mouse cells, Knight et al. (2015) have demonstrated that even if Cas9 search efficiency is reduced in heterochromatic regions, Cas9 is still able to access successfully such regions (Knight et al., 2015).

Chakrabarti et al. have also come to similar conclusions. They observed that upon treatment with the histone deacetylase inhibitor TSA, indel formation is increased suggesting that chromatin decompaction augments Cas9 binding and editing efficiency (Chakrabarti et al., 2019). These results are in line with previous observations arguing for a lower editing efficiency associated with heterochromatin status (Chen et al., 2016; Daer et al., 2017; Kallimasioti-Pazi et al., 2018). In contrast, inhibition of the H3K27me3 methyltransferase EZH2, reduced indel formation, but with a less pronounced impact than TSA treatment. The fact that HDAC inhibition leads to the loss of constitutive heterochromatin and EZH2 inhibition, of facultative heterochromatin, suggests that different types of heterochromatin affect Cas9 editing in distinct ways (Chakrabarti et al., 2019). Nevertheless, these differences might not reflect only direct chromatin changes but indirect alterations on gene expression of DNA repair or other relevant genes. In agreement with this notion, even though both TSA and Ezh2i had an effect on indel formation, the authors were able to observe changes only in chromatin acetylation and not in H3K27me3 methylation. The same study demonstrated differences in ratios of different indels depending on a chromatin context. However, this did not affect dominant indels, suggesting that these changes are minor (Chakrabarti et al., 2019). Such results support the notion that in addition to the sequence around the break, certain chromatin context can modulate editing effectiveness.

Therefore, based on multiple studies with different experimental approaches and systems, we can conclude that chromatin state influences Cas9-mediated genome editing efficiency with heterochromatin being an obstacle for this process. However, indel patterns are mostly unaffected.

## Cas 9 for Knock INS (KIS)

Utilization of the CRISPR-cas9 system for genetic replacement is particularly exciting as it can be implemented in the clinical setting for the cure of genetic diseases. Genetic replacement or KI is mediated by homology-directed repair (HDR).

Several recent studies have investigated the best ways to increase KI potential using Cas9. The most efficient way described so far is incorporation of a single stranded oligonucleotide DNA (ssODN), via single-strand template repair (SSTR). Farboud et al. performed a study in *C. elegans* to determine an efficient strategy to increase knock in efficiency. Their initial goal was to introduce point mutations as it is often required for therapeutic reasons. They used short single-stranded oligonucleotides as a template for recombination matching with the protospacer or with the spacer strand. Interestingly, they found that single nucleotide polymorphism (SNP) insertion was strongly biased toward 5′ or 3′ of the PAM according to the use of the protospacer or the spacer strand (respectively) as a repair template (Farboud et al., 2019). Such polarity can be mainly explained by synthesis-dependent strand annealing (SDSA) mechanism, an HDR pathway in which resected end is annealed to the repair template and extended. After template dissociation the extended end anneals to the other DSB end followed by DNA synthesis to fill the gap (Farboud et al., 2019).

Richardson et al. (2016) also discovered that the binding kinetics of Cas9 with the target DNA is asymmetric. Although Cas9 has a slow release from the template, it releases first the 3’ end of the cleaved DNA strand that is not complementary to the sgRNA (or non-target strand). They observed that the use of an asymmetric donor DNA, complementary to the non-target strand, with 90 nt and 30 nt overlapping the PAM proximal and distal sites respectively, is associated with a higher HDR rate (Richardson et al., 2016). Such findings highlighted the importance for an optimal donor DNA design to ensure high HDR. The same strategy was used to increase HDR efficiency when using ssODN as a donor to correct the β-globin gene (HBB) carrying a mutation responsible for the sickle cell disease (SCD) in human hematopoietic stem/progenitor cells (DeWitt et al., 2016). Another recent study by Okamoto et al. demonstrated the influence of the Cas9 re-cutting capacity of the template DNA on the knock in efficiency using ssODNs. The authors found that either by introducing mutations at the donor sequences that resulted in blocking the re-cutting or either by expressing Cas9/sgRNA transiently using Cas9 protein/sgRNA ribonucleoprotein complexes had a substantial increase on the knock in efficiency (Okamoto et al., 2019).

The use of short single-strand templates was more efficient than a double-strand templates for knock in Farboud et al. (2019). It has recently been demonstrated that in human cells, repair based on a short single-stranded template is Rad51-independent and managed by the Fanconi anemia pathway (Richardson et al., 2018). Thus, differences in efficiencies could be explained by the use of different pathways, and potentially by differential requirements for the length of a template. In the case of a large DNA fragment insertion, the use of a double-stranded template becomes a requirement. For large fragments insertions, Farboud et al. were able to introduce a 9.3 kb fragment by adding a second DSB 340 bp from the initial DSB site. Interestingly, HR efficiency is influenced by the orientation of PAMs. Efficiency was much higher when recognition sites were selected on different strands rather than a single strand. These results suggest that the sequence around the break is important for Cas9-mediated knock in efficiency using larger DNA sequences as donors (Farboud et al., 2019). Insertion efficiency mediated by HR, for DNA fragment as long as 800 bp is also increased after NHEJ inhibition (using Scr7 ligase IV inhibitor treatment) in a bone marrow derived dendritic cell line (DC2.4) (Maruyama et al., 2015). Similarly, SSTR was increased in several genes and cell types when cells were baring a mutation into the human PRKDC gene (encoding for the DNA-PKcs protein) that suppress DNA-PKCs kinase activity (Riesenberg et al., 2019). Promoting homology directed repair (HDR) was also achieved through 53BP1 (a pro-NHEJ factor) inhibition in both human and mouse cells (Canny et al., 2018). This observation might be useful for knock in experimental design.

Since HR takes place during replicative and post replicative stages of the cell cycle, Gutschner et al. developed a system to restrict Cas9 expression to S/G2/M cell cycle phases. By fusing the Cas9 nuclease to geminin they were able to convert Cas9 into a substrate for the APC/Cdh1 complex, which promotes proteins ubiquitination and therefore degradation during late M and G1 phases. In a reporter assay, they monitored HDR-mediated EGFP expression restoration and showed an increase in HDR rate (up to 1.87-fold compare to wt Cas9). They also observed an increase of HDR at a target endogenous locus in HEK293T cells (Gutschner et al., 2016). Along the same lines, delivery of the Cas9/sgRNA ribonucleoprotein complex in cells arrested with nocodazole and aphidicolin and then released, increased SSTR (Lin et al., 2014).

Other groups developed strategies to increase HDR efficiency, allowing spatial proximity between the DSB site and the repair template. By fusing Cas9 to the PCV protein (porcine circovirus 2 rep), forming robust covalent link to a donor DNA, Aird et al. were able to increase HDR efficiency in human cell lines. Using different assays, they showed that covalent tethering of donor DNA template enhances (i) HDR mediated peptide-tag insertion (up to 30-fold) and (ii) HDR mediated mCherry fluorescence restoration (in reporter cells expressing a mutant mCherry) (Aird et al., 2018). Savic et al. came to the same conclusion using snap-tag technology to link donor DNA template to Cas9 and showed that repair template linkage enhances HDR efficiency in a fluorescent reporter cell line and, importantly, also at targeted endogenous loci in K562 and mES cells (Savic et al., 2018).

Another approach to increase HDR efficiency using the Cas9 nuclease fused to CtIP protein (an essential factor promoting DNA end resection) has been described by Charpentier et al. They revealed that tethering CtIP next to the DSB site enhances GFP transgene integration in human fibroblasts. HDR stimulation was also observed in human iPS cells and rat oocytes but depends on the guide RNA (Charpentier et al., 2018).

Chromatin structure has a big influence on homologous recombination (Clouaire et al., 2018; Mitrentsi et al., 2020) but weather it has any influence on Cas9-mediated KI still remains elusive. The expectation is that it will be largely affected by the pre-existing structure of the chromatin surrounding the break. Kallimasioti-Pazi et al. however, found no consistent influence of pre-existing chromatin state on HDR efficiency across several imprinted genes. Systematic analysis on different genomic sites corresponding to different chromatin states will shed more light into the issue.

## Conclusion

In conclusion, genome editing using targeted nucleases, including Cas9, is a complex process, and its success depends on our understanding of specific mechanisms of DSB repair. It has become clear that repair outcome is predominantly sequence-specific and can minimally be altered by other factors. On the other hand, editing efficiency can be influenced by local chromatin structure and therefore can be improved by a change in the chromatin environment.

## Author Contributions

LC and OM collected the literature and wrote the manuscript. ES wrote the manuscript.

## Conflict of Interest

The authors declare that the research was conducted in the absence of any commercial or financial relationships that could be construed as a potential conflict of interest.

## References

[B1] Aird E. J. Lovendahl K. N. St. Martin A. Harris R. S. Gordon W. R. (2018). Increasing Cas9-mediated homology-directed repair efficiency through covalent tethering of DNA repair template. *Commun. Biol.* 1:54. 10.1038/s42003-018-0054-2 30271937 PMC6123678

[B2] Allen F. Crepaldi L. Alsinet C. Strong A. J. Kleshchevnikov V. De Angeli P. (2018). Predicting the mutations generated by repair of Cas9-induced double-strand breaks. *Nat. Biotechnol.* 37 64–82. 10.1038/nbt.4317 30480667 PMC6949135

[B3] Bae S. Kweon J. Kim H. S. Kim J. S. (2014). Microhomology-based choice of Cas9 nuclease target sites. *Nat. Methods* 11 705–706. 10.1038/nmeth.3015 24972169

[B4] Brinkman E. K. Chen T. de Haas M. Holland H. A. Akhtar W. van Steensel B. (2018). Kinetics and fidelity of the repair of Cas9-induced double-strand DNA breaks. *Mol. Cell.* 70 801–813. 10.1016/j.molcel.2018.04.016 29804829 PMC5993873

[B5] Canny M. D. Moatti N. Wan L. C. K. Fradet-Turcotte A. Krasner D. Mateos-Gomez P. A. (2018). Inhibition of 53BP1 favors homology-dependent DNA repair and increases CRISPR-Cas9 genome-editing efficiency. *Nat. Biotechnol.* 36 95–102. 10.1038/nbt.4021 29176614 PMC5762392

[B6] Chakrabarti A. M. Henser-Brownhill T. Monserrat J. Poetsch A. R. Luscombe N. M. Scaffidi P. (2019). Target-specific precision of CRISPR-mediated genome editing. *Mol. Cell.* 73 699–713. 10.1016/j.molcel.2018.11.031 30554945 PMC6395888

[B7] Chang H. H. Y. Pannunzio N. R. Adachi N. Lieber M. R. (2017). Non-homologous DNA end joining and alternative pathways to double-strand break repair. *Nat. Rev. Mol. Cell Biol.* 18 495–506. 10.1038/nrm.2017.48 28512351 PMC7062608

[B8] Charpentier M. Khedher A. H. Y. Menoret S. Brion A. Lamribet K. Dardillac E. (2018). CtIP fusion to Cas9 enhances transgene integration by homology-dependent repair. *Nat. Commun.* 9 1–11. 10.1038/s41467-018-03475-7 29556040 PMC5859065

[B9] Chen X. Rinsma M. Janssen J. M. Liu J. Maggio I. Gonçalves M. A. F. V. (2016). Probing the impact of chromatin conformation on genome editing tools. *Nucleic Acids Res.* 44 6482–6492. 10.1093/nar/gkw524 27280977 PMC5291272

[B10] Chen Y. Zeng S. Hu R. Wang X. Huang W. Liu J. (2017). Using local chromatin structure to improve CRISPR/Cas9 efficiency in zebrafish. *PLoS One* 12:182528. 10.1371/journal.pone.0182528 28800611 PMC5553855

[B11] Ciccia A. Elledge S. J. (2010). The DNA damage response: making it safe to play with knives. *Mol. Cell.* 40 179–204. 10.1016/j.molcel.2010.09.019 20965415 PMC2988877

[B12] Clouaire T. Rocher V. Lashgari A. Arnould C. Aguirrebengoa M. Biernacka A. (2018). Comprehensive mapping of histone modifications at DNA double-strand breaks deciphers repair pathway chromatin signatures. *Mol. Cell* 72 250–262. 10.1016/j.molcel.2018.08.020 30270107 PMC6202423

[B13] Cong L. Ran F. A. Cox L. (2013). Multiplex genome engineering using CRISPR/Cas systems. *Science* 339 819–824. 10.1126/science.1231143 23287718 PMC3795411

[B14] Daer R. M. Cutts J. P. Brafman D. A. Haynes K. A. (2017). The impact of chromatin dynamics on Cas9-mediated genome editing in human cells. *ACS Synthet. Biol.* 6 428–438. 10.1021/acssynbio.5b00299 27783893 PMC5357160

[B15] Decottignies A. (2013). Alternative end-joining mechanisms: a historical perspective. *Front. Genet.* 4:48 10.3389/fgene.2013.00048 PMC3613618 23565119

[B16] DeWitt M. A. Magis W. Bray N. L. Wang T. Berman J. R. Urbinati F. (2016). Selection-free genome editing of the sickle mutation in human adult hematopoietic stem/progenitor cells. *Sci. Trans. Med.* 8:360. 10.1126/scitranslmed.aaf9336 27733558 PMC5500303

[B17] Farboud B. Severson A. F. Meyer B. J. (2019). Strategies for Ef fi cient genome editing. *Genetics* 211 431–457. 30504364 10.1534/genetics.118.301775 PMC6366907

[B18] Gaj T. Gersbach C. A. Barbas C. F. (2013). ZFN, TALEN, and CRISPR/Cas-based methods for genome engineering. *Trends Biotechnol.* 31 397–405. 10.1016/j.tibtech.2013.04.004 23664777 PMC3694601

[B19] Gilbert L. A. Horlbeck M. A. Adamson B. Jacqueline E. Chen Y. Whitehead E. H. (2015). Genome-scale CRISPR-mediated control of gene repression and activation. *Cell* 159 647–661. 10.1016/j.cell.2014.09.029.Genome-Scale PMC4253859 25307932

[B20] Gutschner T. Haemmerle M. Genovese G. Draetta G. F. Chin L. (2016). Post-translational regulation of Cas9 during G1 enhances homology-directed repair. *Cell Rep.* 14 1555–1566. 10.1016/j.celrep.2016.01.019 26854237

[B21] HinzJ. M.LaugheryM. F.WyrickJ. J. (2015). Nucleosomes inhibit Cas9 endonuclease activity in vitro. *Biochemistry* 54 7063–7066. 10.1021/acs.biochem.5b01108 26579937

[B22] Horlbeck M. A. Witkowsky L. B. Guglielmi B. Replogle J. M. Gilbert L. A. Villalta J. E. (2016). Nucleosomes impede cas9 access to DNA in vivo and in vitro. *eLife* 5:12677. 10.7554/eLife.12677 26987018 PMC4861601

[B23] Hsu P. D. Lander E. S. Zhang F. (2014). Development and applications of CRISPR-Cas9 for genome engineering. *Cell* 157 1262–1278. 10.1016/j.cell.2014.05.010 24906146 PMC4343198

[B24] Hsu P. D. Scott D. A. Weinstein J. A. Ran F. A. Konermann S. Agarwala V. (2013). DNA targeting specificity of RNA-guided Cas9 nucleases. *Nat. Biotechnol.* 31 827–832. 10.1038/nbt.2647 23873081 PMC3969858

[B25] Isaac R. S. Jiang F. Doudna J. A. Lim W. A. Narlikar G. J. Almeida R. (2016). Nucleosome breathing and remodeling constrain CRISPR-Cas9 function. *eLife* 5:13450. 10.7554/eLife.13450 27130520 PMC4880442

[B26] Ishino Y. Shinagawa H. Makino K. Amemura M. Nakatura A. (1987). Nucleotide sequence of the iap gene, responsible for alkaline phosphatase isoenzyme conversion in *Escherichia coli*, and identification of the gene product. *J. Bacteriol.* 169 5429–5433. 10.1128/jb.169.12.5429-5433.1987 3316184 PMC213968

[B27] Jansen R. Van Embden J. D. A. Gaastra W. Schouls L. M. (2002). Identification of genes that are associated with DNA repeats in prokaryotes. *Mol. Microbiol.* 43 1565–1575. 10.1046/j.1365-2958.2002.02839.x 11952905

[B28] Jiang F. Doudna J. A. (2017). CRISPR – Cas9 structures and mechanisms. *Annu. Rev. Biophys.* 46 505–531. 28375731 10.1146/annurev-biophys-062215-010822

[B29] Jinek M. Chylinski K. Fonfara I. Hauer M. Doudna J. A. Charpentier E. (2012). A programmable Dual-RNA – guided. *Science* 337 816–822. 10.1126/science.1225829 22745249 PMC6286148

[B30] Kallimasioti-Pazi E. M. Thelakkad Chathoth K. Taylor G. C. Meynert A. Ballinger T. Kelder M. J. E. (2018). Heterochromatin delays CRISPR-Cas9 mutagenesis but does not influence the outcome of mutagenic DNA repair. *PLoS Biol.* 16:2005595. 10.1371/journal.pbio.2005595 30540740 PMC6306241

[B31] Kalousi A. Soutoglou E. (2016). Nuclear compartmentalization of DNA repair. *Curr. Opin. Genet. Dev.* 37 148–157. 10.1016/j.gde.2016.05.013 27266837

[B32] Kim S. I. Matsumoto T. Kagawa H. Nakamura M. Hirohata R. Ueno A. (2018). Microhomology-assisted scarless genome editing in human iPSCs. *Nat. Commun.* 9:3044. 10.1038/s41467-018-03044-y 29507284 PMC5838097

[B33] Knight S. C. Xie L. Deng W. Guglielmi B. Witkowsky L. B. Bosanac L. (2015). Dynamics of CRISPR-Cas9 genome interrogation in living cells. *Science* 350 823–826. 10.1126/science.aac6572 26564855

[B34] Kosicki M. Tomberg K. Bradley A. (2018). Repair of double-strand breaks induced by CRISPR–Cas9 leads to large deletions and complex rearrangements. *Nat. Biotechnol.* 36:4192. 10.1038/nbt.4192 30010673 PMC6390938

[B35] Kuscu C. Arslan S. Singh R. Thorpe J. Adli M. (2014). Genome-wide analysis reveals characteristics of off-target sites bound by the Cas9 endonuclease. *Nat. Biotechnol.* 32 677–683. 10.1038/nbt.2916 24837660

[B36] Lemos B. R. Kaplan A. C. Bae J. E. Ferrazzoli A. E. Kuo J. Anand R. P. (2018). CRISPR/Cas9 cleavages in budding yeast reveal templated insertions and strand-specific insertion/deletion profiles. *Proc. Natl. Acad. Sci. U.S.A.* 115 E2010–E2047. 10.1073/pnas.1716855115 29440496 PMC5834694

[B37] Lin S. Staahl B. T. Alla R. K. Doudna J. A. (2014). Enhanced homology-directed human genome engineering by controlled timing of CRISPR/Cas9 delivery. *eLife* 3:e04766. 10.7554/eLife.04766 25497837 PMC4383097

[B38] Mali P. Yang L. Esvelt K. M. Aach J. Guell M. DiCarlo J. E. (2013). RNA-guided human genome engineering via Cas9. *Science* 339 823–826. 10.1126/science.1232033 23287722 PMC3712628

[B39] Maruyama T. Dougan S. K. Truttmann M. C. Bilate A. M. Ingram J. R. Ploegh H. L. (2015). Increasing the efficiency of precise genome editing with CRISPR-Cas9 by inhibition of nonhomologous end joining. *Nat. Biotechnol.* 33 538–542. 10.1038/nbt.3190 25798939 PMC4618510

[B40] Mitrentsi I. Yilmaz D. Soutoglou E. (2020). How to maintain the genome in nuclear space. *Curr. Opin. Cell Biol.* 64 58–66. 10.1016/j.ceb.2020.02.014 32220808

[B41] O’Geen H. Henry I. M. Bhakta M. S. Meckler J. F. Segal D. J. (2015). A genome-wide analysis of Cas9 binding specificity using ChIP-seq and targeted sequence capture. *Nucleic Acids Res.* 43 3389–3404. 10.1093/nar/gkv137 25712100 PMC4381059

[B42] Okamoto S. Amaishi Y. Maki I. Enoki T. Mineno J. (2019). Highly efficient genome editing for single-base substitutions using optimized ssODNs with Cas9-RNPs. *Sci. Rep.* 9 1–11. 10.1038/s41598-019-41121-4 30886178 PMC6423289

[B43] Richardson C. Ray G. DeWitt M. (2016). Enhancing homology-directed genome editing by catalytically active and inactive CRISPR-Cas9 using asymmetric donor DNA. *Nat. Biotechnol.* 34 339–344. 10.1038/nbt.3481 26789497

[B44] Richardson C. D. Kazane K. R. Feng S. J. Zelin E. Bray N. L. Schäfer A. J. (2018). CRISPR–Cas9 genome editing in human cells occurs via the Fanconi anemia pathway. *Nat. Genet.* 50 1132–1139. 10.1038/s41588-018-0174-0 30054595

[B45] Riesenberg S. Chintalapati M. Macak D. Kanis P. Maricic T. Pääbo S. (2019). Simultaneous precise editing of multiple genes in human cells. *Nucleic Acids Res.* 47 e116. 10.1093/nar/gkz669 31392986 PMC6821318

[B46] Sauer B. (2002). Cre/lox: One more step in the taming of the genome. *Endocrine* 19 221–227. 10.1385/endo:19:3:221 12624421

[B47] Savic N. Ringnalda F. C. A. S. Lindsay H. Berk C. Bargsten K. Li Y. (2018). Covalent linkage of the DNA repair template to the CRISPR-Cas9 nuclease enhances homology-directed repair. *eLife* 7:33761. 10.7554/eLife.33761 29809142 PMC6023611

[B48] Shin H. Y. Wang C. Lee H. K. Yoo K. H. Zeng X. Kuhns T. (2017). CRISPR/Cas9 targeting events cause complex deletions and insertions at 17 sites in the mouse genome. *Nat. Commun.* 8:15464. 10.1038/ncomms15464 28561021 PMC5460021

[B49] Taheri-Ghahfarokhi A. Taylor B. J. M. Nitsch R. Lundin A. Cavallo A. L. Madeyski-Bengtson K. (2018). Decoding non-random mutational signatures at Cas9 targeted sites. *Nucleic Acids Res.* 46 8417–8434. 10.1093/nar/gky653 30032200 PMC6144780

[B50] van Overbeek M. Capurso D. Carter M. M. Thompson M. S. Frias E. Russ C. (2016). DNA repair profiling reveals nonrandom outcomes at Cas9-mediated breaks. *Mol. Cell.* 63 633–646. 10.1016/j.molcel.2016.06.037 27499295

[B51] Wang F. Qi L. S. (2016). Applications of CRISPR genome engineering in cell biology. *Trends Cell Biol.* 26 875–888. 10.1016/j.tcb.2016.08.004 27599850 PMC5077632

[B52] Wu X. Scott D. A. Kriz A. J. Chiu A. C. Hsu P. D. Dadon D. B. (2014). Genome-wide binding of the CRISPR endonuclease Cas9 in mammalian cells. *Nat. Biotechnol.* 32 670–676. 10.1038/nbt.2889 24752079 PMC4145672

